# Epidemiology and Risk Factors of Alzheimer’s Disease in Iran: A Systematic Review

**Published:** 2019-12

**Authors:** Elham NAVIPOUR, Mahbobeh NEAMATSHAHI, Zahra BARABADI, Mohammad NEAMATSHAHI, Aghilallah KEYKHOSRAVI

**Affiliations:** 1. Department of Biostatistics, Faculty of Paramedicine, Sabzevar University of Medical Sciences, Sabzevar, Iran; 2. Department of Community Medicine, Faculty of Medicine, Research Center Social Determinants Health, Sabzevar University of Medical Sciences, Sabzevar, Iran; 3. Department of Physiology and Pharmacology, School of Medicine, Sabzevar University of Medical Sciences, Sabzevar, Iran; 4. Department of Anesthesiology, Faculty of Medicine, Sabzevar University of Medical Sciences, Sabzevar, Iran; 5. Department of Pediatrics, Faculty of Medicine, Sabzevar University of Medical Sciences, Sabzevar, Iran

**Keywords:** Epidemiology, Risk factors, Alzheimer, Dementia, Iran

## Abstract

**Background:**

Alzheimer’s disease is a chronic disease characterized by a progressive decline in mental abilities and quality of life alongside behavioral abnormalities associated with high economic burden. The purpose of this study was to investigate epidemiology and risk factors of Alzheimer’s disease in Iran.

**Methods:**

In this systematic review study, both Persian and English-language databases including Medline, Google Scholar, PubMed, web of science and Magiran were searched using following keywords: epidemiology, Alzheimer, dementia and Iran without time limit up to 2017. Thirty articles abstract out of 50 studies related to this topics, were reviewed. Of which 12 full text entered into the quality assessment process and finally, four articles were selected for inclusion in this study and their results was extracted.

**Results:**

The total sample size of the 4 selected studies was 2781. The prevalence of Alzheimer’s disease in the current study was estimated to be 2.3% in the population of 67–78 years old. Age, genetics, depression and hypertension were determined as the risk factors for Alzheimer’s disease, while daily listening to music, meeting weekly with friends and daily intake of vitamin E were considered as the factors with protective role in this disease.

**Conclusion:**

Alzheimer’s disease is one of the main causes of functional dependence and mortality in the elderly people. Lifestyle changes and multiple mental activities in elderly increases the cognitive ability of these population, which will reduce direct and indirect costs of this disease.

## Introduction

Population aging is accompanied by the increasing rate of age-related diseases. World widely, 82% of the elderly people suffer from at least one chronic disease while 65% suffer from more than one (1).

Alzheimer’s disease (AD) is a chronic disorder causing disability in the elderly, with an upward trend in the last century (2). It is the most common cause of dementia in the last century, accounting for 75% of all dementia cases. Currently, more than 35 million people suffer from AD in developed and developing countries (3). Approximately 5.5 million people in US are affected by this disease. The number of people diagnosed with AD is predicted to double in every 20 years by the end of 2040. As the world’s elder population grows, the number of people at risk increases and imposes high direct and indirect costs (4). In 2005, a group of international experts commissioned by International Alzheimer’s Association, estimated the prevalence of Alzheimer’s in 14 world regions based on epidemiological data. The results of the survey showed that 4.6 million new cases occur each year, which is influenced by the geographical location (5), with the highest incidence in North America and Western Europe at the age of 60. The annual incidence rate of AD (per 1000 people) was estimated to be 10.5 in North America and 8.8 in Western Europe. Elevated number of people affected with AD is the consequence of increasing survival rate up to age 80 or above (4).

In Iran, as well as the other countries the aging population is growing. Since, there is a strong association between AD and aging, it is expected that 8–10% of the elderly people in Iran will be affected by this disease over the next 2–3 decades (6). People over the age of 60 suffering from AD spend around 11.2% of their lives with disability. The total cost of medicine and health care for affected people with AD is three times greater than that of other people (3). On the other hand, since there is no effective treatment for this disease today, prevention and lifestyle modification are critical (7).

AD is the most common type of dementia and the most important cognitive disorder (8), which usually begins with impaired short-term memory and finally, it affects all intellectual functions and leads to a complete dependence in daily activities and premature death (9). The main cause is unknown, but age, genetic and some non-genetic factors play major roles in the progression of the disease (4). It is difficult to diagnose in early stages, because subjects refer to medical centers while their cognitive skills have significantly impaired (10–12).

Alzheimer’s prevalence in developing and developed countries has increased due to mortality due to malnutrition, inflammatory diseases, war, poverty and infectious diseases (13). Aging population is considered as a global phenomenon (14) with highest rate in developing countries (15). As the number of people diagnosed with AD increases with age (16, 17), economic burden on the health care system is also expected to increase in the coming years (4).

Since there has been no systematic review that collect and review all relevant studies in this field; addressing this issue could be helpful in designing effective intervention strategies for disease control. So, the aim of this systematic review was to investigate the epidemiology and risk factors of AD in Iran.

## Methods

### Search strategy

The present study is a systematic review on the epidemiology and risk factors of AD in Iran. The literature search was conducted in PubMed, MEDLINE, Web of Science, Scopus, Google Scholar, Springer, Cochrane library, SID and Magiran for both English and Persian-language articles and abstracts as well as reference list of the relevant articles and congresses’ papers, without time limitation. Key words used in the search was obtained from Mesh and included epidemiology, risk factors, Alzheimer, Iran, Dementia.

#### Selection Criteria

At first, a list of relevant topics and abstracts was independently prepared by two reviewers. After repetitive titles removal, retrieved abstracts was reviewed to find related articles. Inclusion criteria for this study was case-control and cross-sectional studies with relevance to Alzheimer’s epidemiology and related risk factors in Iran. Minimum entry criteria were applied to increase the sensitivity of article selection. However, in order to achieve the most relevant and high quality papers, exclusion criteria were as follows: non-relevant articles in terms of type of study, low-quality studies based on CASP checklist and studies that did not contain enough information.

#### Evaluation of articles quality

Articles quality were evaluated using a CASP checklist by two researchers. This checklist contains 11 sections for cross-sectional studies and case-control studies, which are scored in each section based on points in the checklist.

#### Data extraction:

In the full text review, studies without sufficient information as well as articles without full text available were excluded from the study. The quality of the finally selected studies were evaluated using CASP checklist. The information extracted from these articles included the first author’s name, the country and area where the study was conducted, publication time of article, sample size, and data collection method (Tables 1 and 2).

**Table 1:** Characteristics of four studies that were eligible for entry into this structured review

***Ref. No.***	***Date of publication***	***Country***	***Age (yr)***	***Participants***	***Information gathering location***
(18)	2004	Iran-Tehran	75± 10 73.5±11.5	105 AD 129 control	Tehran-Iran
(19)	2008	Iran	70.6±8.18 and 69.4±7.92	115 AD 115 control	Iran
(20)	2012	Iran	78.55 ± 7.80 77.14 ± 6.95	156 AD 161 control	Iran
(21)	2017	Iran	67.4±6.44	2000 elderly	Iran

**Table 2:** Methodology of the four studies selected for entering in this structured review

***Ref. No.***	***Study design***	***Statistical methods***	***Outcome***	***Exposure***	***Measure of association (95%CI)***	***Conclusions***
(18)	Case-control study	χ^2^ test	Alzheimer disease	APOE4	OR	The APOE allele increase the risk for AD in dose and age dependent manner in a population from Tehran Iran
(19)	case-control study	Pearson’s χ^2^-test Mann-Whitney test	Alzheimer’s Disease	Age Hypertension	OR	These results confirm the previously reported relationship between AD and vascular factors
(20)	Case-control	χ^2^ test	Alzheimer disease	CCR2 and CCR5	OR	Our study did not show an association between CCR5Δ32 and CCR2-64I variations and AD in the Iranian population. OR=1.1 (0.4–3.1) & 1.27 (0.73–2.2)
(21)	Cross sectional study	Logistic regression	Alzheimer’s disease	Depression	Prevalence	The prevalence of Alzheimer disease was 2.3%. The logistic regression indicated that depression was one of the risk factors for Alzheimer in the elderly. The other diseases such as heart diseases, diabetes, thyroid dysfunction, arthritis, migraine, hyperlipidemia, and cancer had no effects on the development of Alzheimer in the subjects of the current study.

**Fig. 1:**
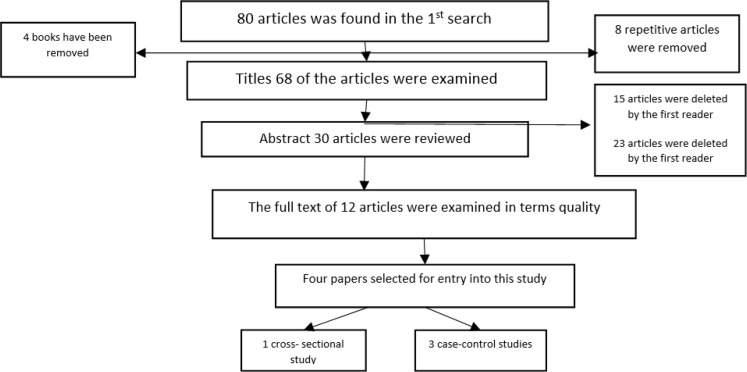
Flow chart the process of study entering in a systematic review

In the first step, a total of 80 articles were retrieved by searching databases and bibliographies. 50 papers were excluded because they did not meet appropriately the inclusion criteria. Upon reviewing 30 relevant abstracts, the full text of 12 articles were entered into the quality assessment process. 4 papers were finally selected for this study. Of these four articles, one was cross-sectional and three case-control studies (Fig. 1).

## Results

Among all retrieved papers, 4 studies met inclusion criteria. The diagram shows the selection process of papers entered the study (Fig. 1). The quality assessment of 12 papers was performed using the CASP checklist, of which 4 articles were finally included in the present study.

The total sample size of these 4 studies was 2781. All of these studies were performed on both sexes within a mean age of 72 years.

In another study, APOE allele increased the risk of Alzheimer in Tehran’s population (18). Foroughan et al, found a relationship between AD and vascular diseases such as hypertension and no relationship with positive family history, history of depression, head trauma, multi-drug consumption, sedentary life style (physical and social activity), diabetes and smoking with AD (19).

No significant difference was detected in the distribution of Ccr2-64i and Ccr532 genes between affected people with AD and healthy people in Kamali et al study (20). The prevalence of AD in Iran was estimated 2.3% and depression was a risk factor for AD (21).

There was no relationship between AD and other diseases such as heart diseases, diabetes, thyroid dysfunction, arthritis, migraine, hyperlipidemia and cancers, while daily listening to music, meeting weekly with friends and daily intake of vitamin E were considered as protective factors for this disease. On the other hand, there was no statistically relationship between smoking, using hubble-bubble (hookah), alcohol consumption, drinking coffee and tea, taking vitamin D and narcotics with AD (21).

### Epidemiology

AD is a critical public health issue in the United States and many other countries around the world, with a significant health, social, and financial burden on society. Worldwide, it is estimated that 35 million people have AD or other types of dementia, and about 65 million people are expected to have dementia by 2030.

AD is a multi-factorial disease. Many risk factors are involved in its incidence and progression, some of these are modifiable, and others are not. Age is the main risk factor for its development. The probability of this disease is doubled every 5 years over the age 65 (22). 95% of all cases diagnosed with AD are aged over 65 yr categorized under late-onset or sporadic AD and the others which result from a rare genetic mutation, occurring before age 65, identified as early onset or familial AD (23). An autosomal dominant mutation in the genes located on chromosomes 1 and 14 or mutation in the amyloid precursor protein (APP) gene on chromosome 21 are the main known genetic factors associated with familial AD. However, more complex genetic factors are involved in the sporadic forms of AD. One of the identified genetic factors associated with sporadic AD is the epsilon four allele of the Apo lipoprotein E (APOE) gene (24). The longer life expectancy in females results in higher prevalence of AD in this population (25).

The prevalence of AD in the current study was estimated to be 2.3% in the Iranian population of 67–78 years old. Age, ApoE allele, depression and hypertension were identified as the main risk factors associated with this disease in Iran, in this systematic review.

## Discussion

This systematic review was conducted with the aim of evaluating the epidemiology and identifying the risk factors of AD in Iran. Of four papers included in this study, one has investigated the association between lifestyle and AD, while the others have focused on genetic and risk factors associated with this disease. Meta-analysis was not performed in the present study because of insufficient number of articles and heterogeneity in the results.

A review study reported the mean age-adjusted prevalence estimate for dementia among people aged 65 years and older living in developing countries, derived from data published within the past 10 years, was calculated to be 5.3% (17), but, unfortunately, due to the heterogeneity of studies and the inability to report the prevalence in case-control studies, there was no possibility to estimate the prevalence of Alzheimer. On the other hand, most of the elder population at the risk of Alzheimer, which usually die for other reasons, have no chance of progression of AD.

Most Alzheimer’s patients in the Iranian community are being cared for at home due to family dependency and there is no access to all affected people with AD. Therefore, we were unable to make an accurate estimate of the prevalence of AD in Iran. Other factors affecting the estimation on the prevalence of AD are the presence of sample structure based on regional (26). So the prevalence in an area is not an appropriate estimate for the entire community.

The prevalence of AD in Iran estimated by the present study is 3.2%. The highest prevalence of Alzheimer, especially at age 60 has been reported for North America and Western Europe (%6.4 and 5.4% respectively) followed by Latin America (4.9%) and China and its developing western-Pacific neighbors (4.0%) (5). Prevalence of AD in Iran is lower than these countries.

The main risk factors associated with AD in Iranian population are age, ApoE allele, depression and hypertension. Older age and ApoE allele are not-modifiable risk factors but hypertension can be improved with changing life style. Cerebrovascular disease, hypertension, type II diabetes, body weight, smoking and traumatic brain injury increase the risk of AD (4). Another study indicated age as the most important risk factor for AD. Genetic factors, vascular diseases, hypertension, heart disease, cerebrovascular diseases, diabetes, obesity, hyperlipidemia, metabolic syndrome, alcohol, diet, smoking, mental disorder, social economic status, education, physical activity and depression are among of known risk factors for AD (6). There was a significant relationship between diabetes type2, smoking, over-weight and obesity, physical inactivity, hypertension, hypercholesterolemia and AD (27). Also, depression has been identified as another risk factor for AD. The role of other underlying diseases such as frailty, carotid atherosclerosis, hypertension, low diastolic blood pressure, type 2 diabetes mellitus (in Asian population), Low education level, high body mass index (BMI) in middle aged, current smoking (in western population), light-to-moderate drinking, stress was also investigated in the development of AD in this study (28). Age, Apo lipoprotein E, family history, mild cognitive impairment, cardiovascular disorders, education, social and cognitive engagement and traumatic brain injury were found as other risk factors (3). Alzheimer’s family history in first-degree relatives and a history of head injury with loss of consciousness were also risk factors (29).

The limitation of our study was that there was only few studies regarding epidemiology and risk factors of AD in Iran.

## Conclusion

The demographic transition in Iran and an increase in the prevalence of elderly population as a result indicate the importance of increasing the prevalence of diseases associated with aging (including dementia) and its related consequences in this country. AD is one of the main cause of functional dependence and mortality in the elderly people.

The multiple mental activities in elderly and lifestyle changes increases the cognitive ability of the elderly, which will prevent direct and indirect costs of Alzheimer’s.

## Ethical considerations

Ethical issues (Including plagiarism, informed consent, misconduct, data fabrication and/or falsification, double publication and/or submission, redundancy, etc.) have been completely observed by the authors.
